# YAP-dependent ubiquitination and degradation of β-catenin mediates inhibition of Wnt signalling induced by Physalin F in colorectal cancer

**DOI:** 10.1038/s41419-018-0645-3

**Published:** 2018-05-22

**Authors:** Chen Chen, Dongrong Zhu, Hao Zhang, Chao Han, Guimin Xue, Tianyu Zhu, Jianguang Luo, Lingyi Kong

**Affiliations:** Jiangsu Key Laboratory of Bioactive Natural Product Research and State Key Laboratory of Natural Medicines, China Pharmaceutical University, 24 Tong Jia Xiang, Nanjing, 210009 China

## Abstract

Aberrant activation of Wnt/β-catenin signalling is critical in the progression of human cancers, especially colorectal cancer (CRC). Therefore, inhibition of Wnt/β-catenin signalling is a significant potential target for CRC therapy. Here, we identified for the first time that Physalin F (PF), a steroid derivative isolated from *Physalis angulate*, acts as an antagonist of Wnt/β-catenin signalling. In vitro, PF decreased Wnt3a-induced TOPFlash reporter activity in HEK293T cells and promoted the formation of the β-catenin destruction complex. Importantly, PF also inhibited Wnt/β-catenin signalling and accelerated the degradation of β-catenin in CRC cells. However, PF did not affect the stabilization of Axin or the interaction of β-catenin with E-cadherin. Interestingly, we further found that PF promoted YAP binding to the β-catenin destruction complex, which facilitated the ubiquitination and degradation of β-catenin. Silencing and pharmacological inhibition of YAP reversed the formation of the β-catenin destruction complex induced by PF, implying that YAP binding to the β-catenin destruction complex was responsible for PF-mediated inhibition of Wnt/β-catenin signalling. Furthermore, PF observably inhibited tumour growth by down-regulating β-catenin in tumour-bearing mice. Collectively, our findings indicated that PF inhibited Wnt/β-catenin signalling by accelerating the ubiquitination and degradation of β-catenin in a YAP-dependent manner and therefore PF could be a novel potential candidate for CRC therapy.

## Introduction

Colorectal cancer (CRC) is a major cause of cancer-related morbidity and mortality worldwide^1^. Due to advances in surgery and chemotherapy, patients’ overall survival has increased. However, there have emerged severe toxicities and side effects during long-term administration^2^. Therefore, it is urgently necessary to develop novel therapeutic agents for CRC therapy.

The Wnt/β-catenin signalling pathway is crucial in multiple developmental events during embryogenesis and is also involved in tumourigenesis^3, 4^. Aberrant activation of Wnt/β-catenin signalling is frequently observed in CRC and is considered to be a crucial driver of CRC pathogenesis^5^. In the absence of the Wnt ligand, cytoplasmic β-catenin is phosphorylated at residues Ser45, Thr41, Ser37, and Ser33 in a destruction complex with adenomatous polyposis coli (APC), casein kinase 1 (CK1), glycogen synthase kinase-3β (GSK-3β) and Axin^6^. Phosphorylated β-catenin is recognized by the E3 ubiquitin ligase β-transducin repeat-containing protein (β-TrCP) and subsequently degraded by the ubiquitin-dependent proteasome pathway. Upon Wnt stimulation, the Wnt ligand binds to the Frizzled receptor and the LDL receptor related protein (LRP) complex at the cell surface, which leads to the membrane recruitment and activation of scaffold protein and dishevelled^6^. Activated dishevelled inactivates the destruction complex in the cytoplasm, thus decreasing the degradation of β-catenin. The stabilized β-catenin translocates to the nucleus and interacts with TCF/LEF transcription factors to activate Wnt target genes and promote the process of cancer^7^. Therefore, inhibition of β-catenin is a potential strategy for the prevention or treatment of CRC^8–10^.

The transcriptional co-activator Yes-associated protein (YAP), identified as a target of the Hippo pathway has recently been identified as an additional regulatory component of canonical Wnt/β-catenin signalling^11, 12^. In the cytoplasm, YAP interacts with β-catenin directly and restricts nuclear translocation of β-catenin^13^. Moreover, YAP is essential for β-TrCP recruitment to the destruction complex and facilitates β-catenin degradation^14^, indicating that YAP is pivotal for the ubiquitination and proteasomal degradation of β-catenin.

Natural products have recently been gaining more attention because of their multiple biological activities and desirable health benefits, especially in cancer therapy^15, 16^. Withanolides, a class of steroid compounds, have attracted attention due to their multiple bioactivities, such as immunosuppressive, anti-inflammation, antimicrobial, antidiabetic and anti-tumour activities^17, 18^. Our previous studies have also reported the anti-tumour efficacy of some withanolides^19, 20^. Physalin F (PF), a withanolide derivative extracted from *Physalis angulata*, has exhibited immunomodulatory and anti-tumour activities^21–23^. However, there have been no reports on the therapeutic property of PF towards CRC to date. In the present study, we demonstrated for the first time that PF suppressed Wnt/β-catenin signalling by promoting YAP-mediated ubiquitination and proteasomal degradation of β-catenin in CRC cells.

## Results

### Identification of PF as an inhibitor of Wnt/β-catenin signalling

**Fig. 1:**
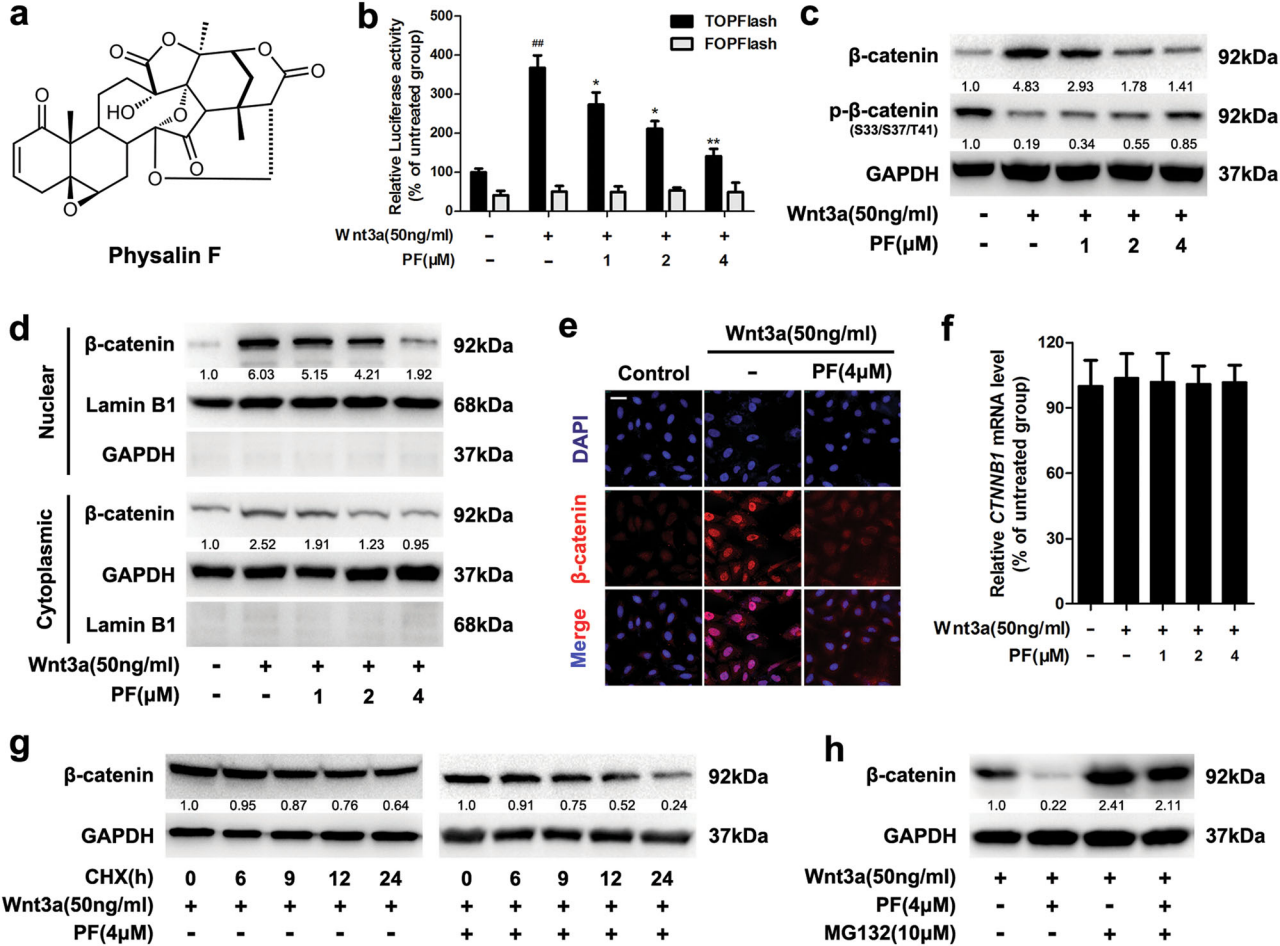
PF inhibited the Wnt/β-catenin signaling. **a** The chemical structure of Physalin F (PF). **b** Luciferase reporter activity was measured in HEK293T cells. After 24 h of transfection, HEK293T cells were treated with recombinant Wnt3a in the presence of PF at the indicated concentrations for 16 h, then followed by a luciferase assay. Data represent the means ± SD (*n* = 3), *P < 0.05, **P < 0.01, compared with recombinant Wnt3a group, ^##^*P* < 0.01, compared with untreated group. **c** HEK293T cells were treated with indicated concentrations of PF in the presence of Wnt3a for 16 h, then the β-catenin and p-β-catenin were detected by immunoblotting. **d** HEK293T cells were treated with indicated concentrations of PF in the presence of Wnt3a for 16 h, then the cytoplasmic and nuclear fractions lysates were analysed by immunoblotting. **e** HEK293T cells were treated with 4 μM PF and Wnt3a for 16 h, then the intracellular expression of β-catenin was examined by immunofluorescence, scale bars = 20 μm. **f** HEK293T cells were treated with indicated concentrations of PF and Wnt3a for 16 h, and the mRNA level of CTNNB1 was measured by quantitative RT-PCR. Data represent the means ± SD (*n* = 3). **g** HEK293T cells were co-treated with or without 4 μM PF and CHX (50 μg/ml) in the presence of Wnt3a for the indicated time intervals, and the expression of β-catenin was examined by immunoblotting. **h** HEK293T cells were treated with 4 μM PF for 12 h, then with or without 10 μM MG132 for an additional 8 h before immunoblotting

To investigate whether PF (Fig. 1a) could inhibit Wnt/β-catenin signalling, we applied a β-catenin/TCF-dependent luciferase reporter (Top-Luc) to evaluate the effect of PF on Wnt/β-catenin signalling in HEK293T cells. The results showed that PF decreased TOPFlash reporter activity induced by Wnt3a recombinant protein (Fig. 1b). In contrast, the activity of FOPFlash (a negative control reporter with mutated β-catenin/TCF binding sites) was unaffected. Correspondingly, treatment with PF resulted in down-regulated expression of β-catenin stimulated by Wnt3a recombinant protein (Fig. 1c). The Wnt3a-induced expression of Wnt/β-catenin downstream target proteins Cyclin D1, c-Myc, and LEF1 was also reduced after treatment with PF (Supplementary Figure S1a and b).

Furthermore, PF suppressed the Wnt3a-induced expression of β-catenin in the nuclear and cytoplasmic fractions (Fig. 1d). Immunofluorescence staining confirmed the reduction of nuclear β-catenin accumulation stimulated by Wnt3a (Fig. 1e). Nevertheless, the mRNA level of CTNNB1, which encodes β-catenin, remained unchanged (Fig. 1f). Notably, after treatment with cycloheximide (CHX, inhibitor of de novo protein synthesis), PF decreased the half-life of β-catenin protein in the presence of Wnt3a (Fig. 1g). These data suggested that PF specifically targeted Wnt/β-catenin signalling by reducing β-catenin protein stability rather than repressing gene expression.

Since the level of intracellular β-catenin is regulated by the proteasomal degradation pathway, we examined the involvement of the proteasome pathway in PF-mediated inhibition of β-catenin. As shown in Fig. 1h, the PF-induced reduction of β-catenin was abrogated by MG132 (a proteasome inhibitor). Similar results were obtained after treatment with the other two proteasome inhibitors ALLN and bortezomib (BTZ) (Supplementary Figure S1c). Moreover, PF elevated the phosphorylation of β-catenin at Ser33/37/Thr41 residues in the presence of Wnt3a (Fig. 1c), which is required for the degradation of β-catenin. These results indicated that PF inhibited Wnt/β-catenin signalling by increasing proteasome-mediated β-catenin degradation.

### PF accelerated the ubiquitination and proteasomal degradation of β-catenin

**Fig. 2:**
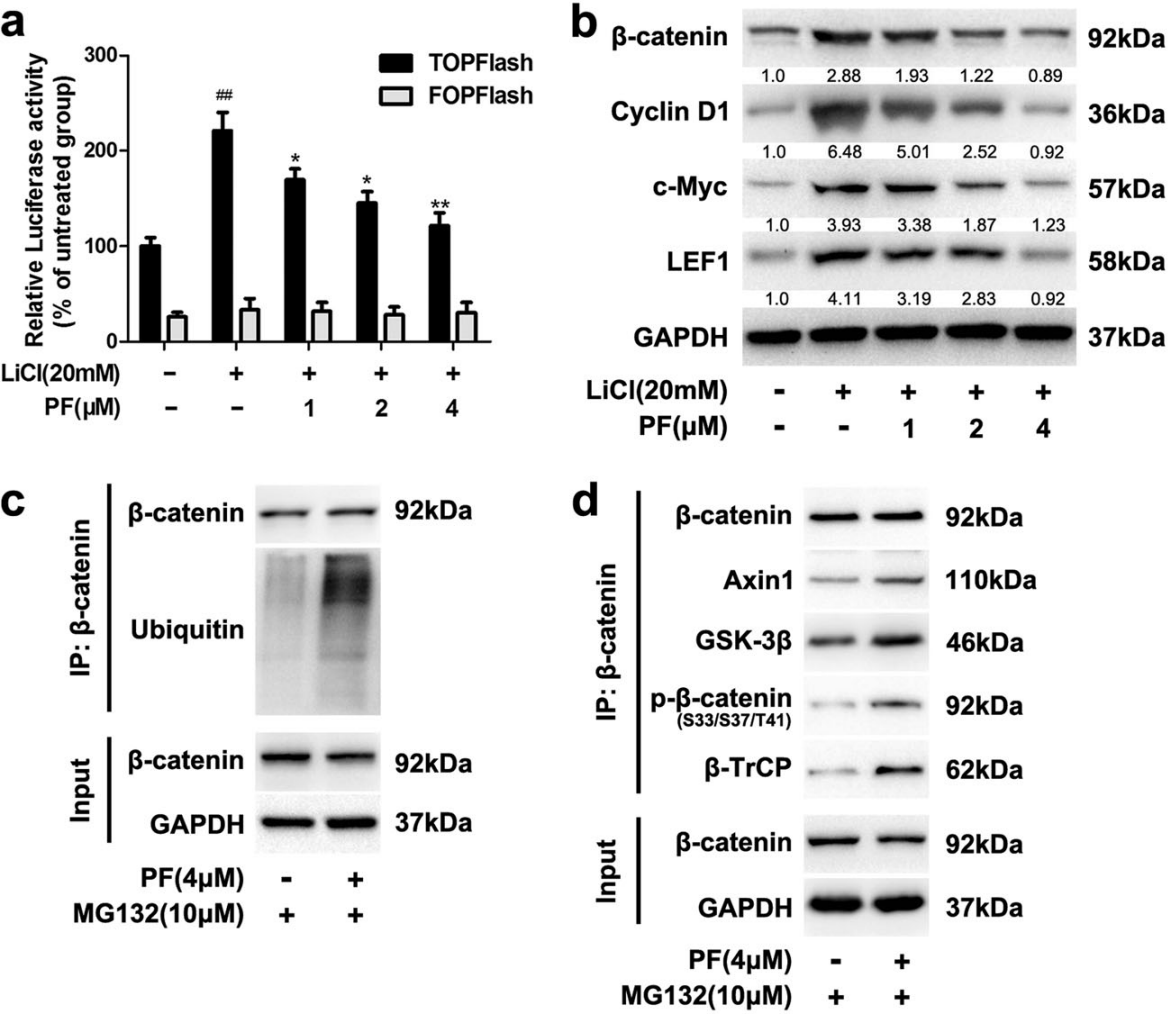
PF promoted the degradation of β-catenin. **a** HEK293T cells were incubated with PF (1, 2 and 4 μM) in the presence of LiCl (20 mM). After 16 h, luciferase activity was determined. Data represent the means ± SD (*n* = 3), *P < 0.05, **P < 0.01, compared with LiCl group, ^##^*P* < 0.01, compared with untreated group. **b** The protein levels of β-catenin and target proteins were observed after treated with different concentrations of PF and LiCl (20 mM) for 16 h. **c**, **d** HEK293T cells were treated with 4 μM PF for 12 h, followed with or without 10 μM MG132 for additional 8 h. β-catenin was immunoprecipitated with a β-catenin antibody. Input and immunoprecipitated fractions were analysed by immunoblotting

In Wnt/β-catenin signalling, GSK-3β catalyses β-catenin phosphorylation at N-terminal residues, which plays an important role in the proteasomal degradation of β-catenin^6^. Thus, we assessed whether GSK-3β participated in PF-induced suppression of β-catenin. After incubation with the known GSK-3β inhibitor LiCl, TOPFlash reporter activity was increased, while this stimulation was suppressed by PF (Fig. 2a). Western blot analysis consistently showed that PF inhibited the protein levels of β-catenin and the target genes stimulated by LiCl and 6-bromoindirubin-3-oxime (BIO, a GSK-3β inhibitor) (Fig. 2b and Supplementary Figure S1d), suggesting that there may be other regulatory factors involved in the inhibition of β-catenin mediated by PF.

As β-catenin is regulated by cytoplasmic destruction complex components, such as Axin, APC and GSK-3β. The destruction complex regulates the sequential phosphorylation of β-catenin, which leads to the polyubiquitination of β-catenin by β-TrCP and subsequent degradation by the proteasome pathway. To determine the ubiquitination level of β-catenin under the influence of PF, we treated HEK293T cells with PF and the proteasome inhibitor MG132. As shown in Fig. 2c and Supplementary Figure S1e, treatment with PF increased the ubiquitination of β-catenin. More importantly, PF enhanced the binding of β-catenin with Axin1, GSK-3β and β-TrCP (Fig. 2d and Supplementary Figure S1f). These data indicated that PF promoted the formation of the β-catenin destruction complex and enhanced the degradation of β-catenin.

### PF inhibited the activity of β-catenin in CRC cells

**Fig. 3:**
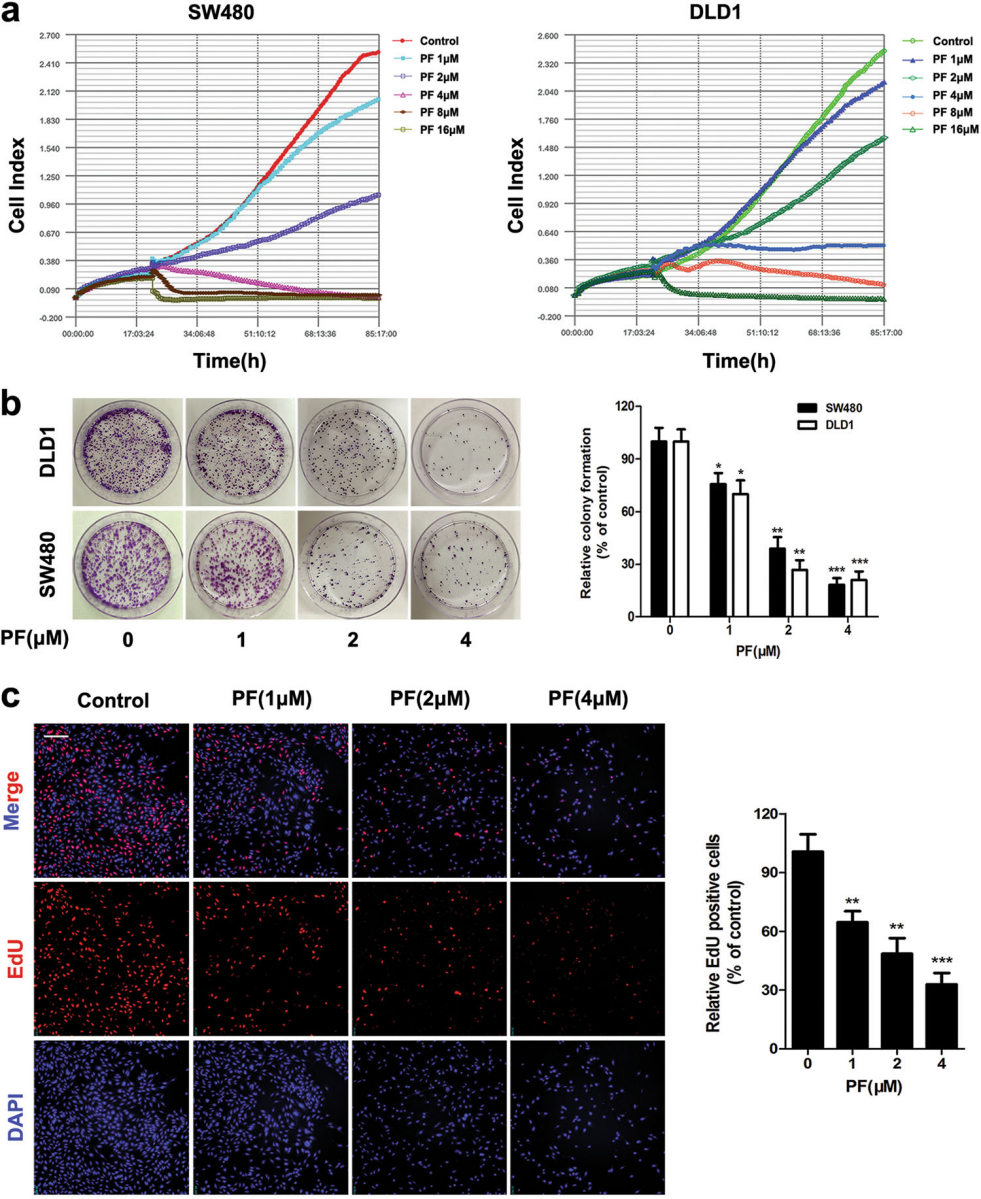
PF suppressed the proliferation of CRC cells. **a** SW480 and DLD1 cells were cultured in E-plates for 20 h, and then treated with different concentrations of PF. Cell proliferation was monitored continuously by using an xCELLigence RTCA system. **b** SW480 and DLD1 cells were treated with different concentrations of PF for 14 days, and colony formation was assessed by staining with crystal violet. Data represent the means ± SD (*n* = 3), *P < 0.05, **P < 0.01 and ***P < 0.001, compared with 0 μM group. **c** After treatment with indicated concentrations of PF in SW480 cells for 24 h, EdU labelling assay was performed and the cells were were observed by ImageXpress® Micro Confocal. Data represent the means ± SD (*n* = 3), **P < 0.01, ***P < 0.001, compared with 0 μM group, scale bars = 50 μm

**Fig. 4:**
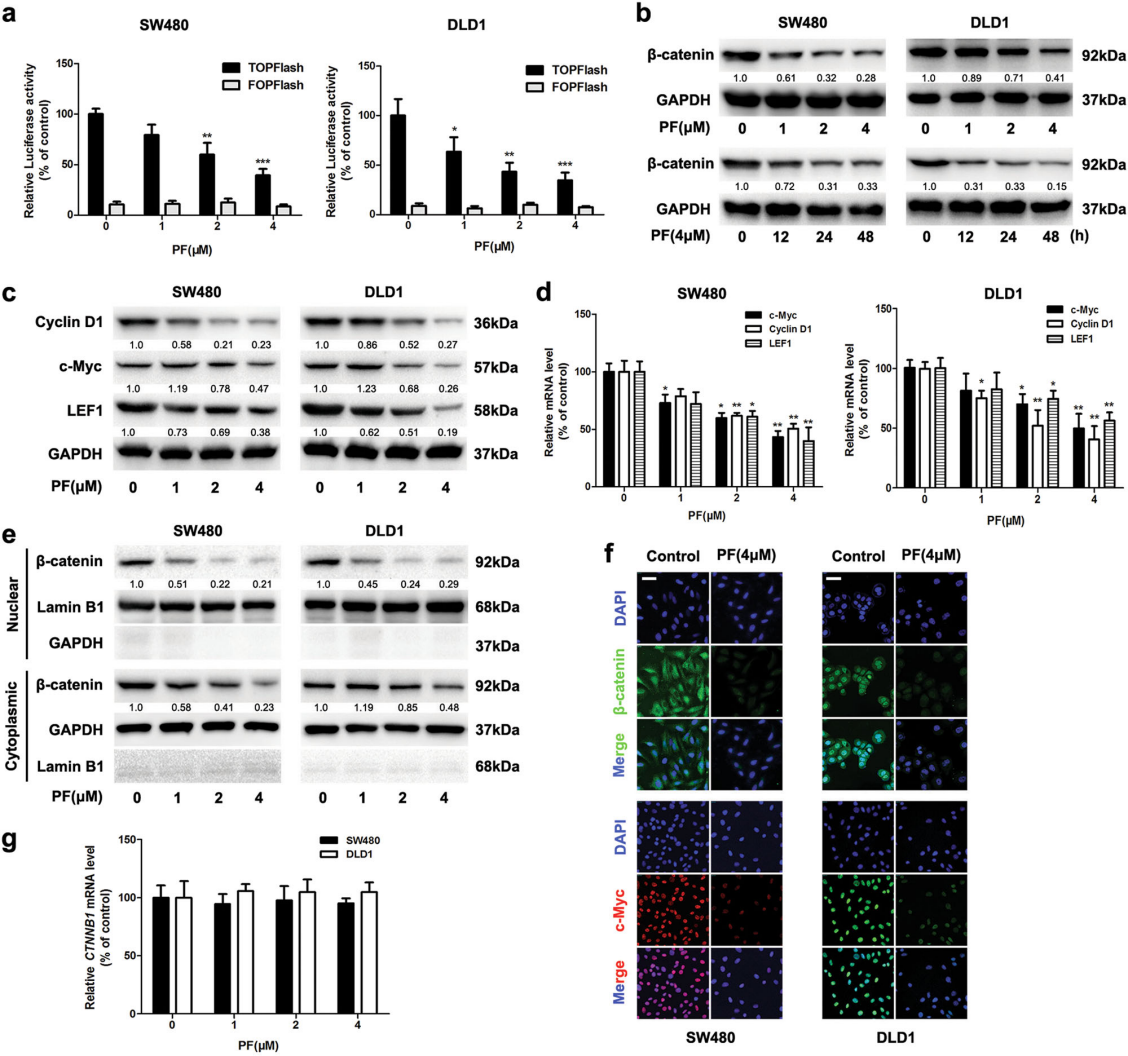
PF inhibited the Wnt/β-catenin Signaling in CRC cells. **a** SW480 and DLD1 cells were transfected with reporter plasmid. After 24 h incubation, cells were treated with PF at the indicated concentrations, then the luciferase activity was measured by the dual luciferase reporter assay system. Data represent the means ± SD (*n* = 3), *P < 0.05, **P < 0.01 and ***P < 0.001, compared with 0 μM group. **b** SW480 and DLD1 cells were treated with different concentrations of PF for 24 h and 4 μM PF for different time intervals. Then the expression of β-catenin was determined by western blot. **c** SW480 and DLD1 cells were treated with different concentrations of PF for 24 h, then the expression of Wnt/β-catenin downstream target proteins cyclin D1, c-Myc, and LEF1 were examined by western blot. **d** After treatment with different concentrations of PF for 24 h, the mRNA expression of Wnt/β-catenin downstream target proteins cyclin D1, c-Myc, and LEF1 were analysed by quantitative RT-PCR. Data represent the means ± SD (*n* = 3), *P < 0.05, **P < 0.01, compared with 0 μM group. **e** SW480 and DLD1 cells were treated with PF for 24 h, then the cytoplasmic and nuclear fractions lysates were analysed by immunoblotting. **f** SW480 and DLD1 cells were treated with 4 μM PF for 24 h, and then the intracellular expression of β-catenin and c-Myc were analysed by immunofluorescence, scale bars = 20 μm. **g** The mRNA level of CTNNB1 was measured by quantitative RT-PCR after treatment with PF for 24 h in SW480 and DLD1 cells. Data represent the means ± SD (*n* = 3)

Evidence is emerging in support of the concept that aberrant activation of β-catenin is oncogenic and is a critical driver in the pathogenesis of CRC^8, 9, 24^. Therefore, targeting β-catenin has been identified as a potential strategy for CRC therapy. We next examined whether PF inhibited the Wnt/β-catenin pathway in SW480 and DLD1 colon cancer cells. Real-time growth kinetics analysis showed that PF inhibited the proliferation of SW480 and DLD1 cells (Fig. 3a). Colony formation of SW480 and DLD1 cells was also reduced after treatment with PF (Fig. 3b). Correspondingly, MTT and EdU labelling assays also revealed that the cell viability of SW480 and DLD1 cells was suppressed by PF (Fig. 3c and Supplementary Figure S2a and b). In accordance with the results in HEK293T cells, PF inhibited the TOPFlash activity in SW480 and DLD1 cells (Fig. 4a). After treatment with the specific inhibitors of Wnt/β-catenin signaling (IWR-1, VAX-939), the inhibition effects of PF on the cell viability of SW480 and DLD1 cells had no significant difference with inhibitors treatment groups (Supplementary Figure S2d). Consistent results were also obtained in TOPFlash activity assay (Supplementary Figure S2e). These results indicated that the inhibitory effects of PF on CRC cells was attributed to Wnt/β-catenin signalling. In addition, we observed that the protein levels of β-catenin were reduced upon treatment with PF (Fig. 4b). The mRNA and protein expression of Wnt/β-catenin downstream targets, including Cyclin D1, c-Myc, and LEF1, was also decreased in response to PF treatment (Fig. 4c, d).

As β-catenin nuclear translocation is a vital process of Wnt/β-catenin signalling^7^, we examined the effect of PF on nuclear translocation of β-catenin. Immunoblotting analysis indicated that PF treatment resulted in a decreased level of β-catenin in the nuclear fractions (Fig. 4e). Furthermore, immunofluorescence analysis visually revealed that PF reduced β-catenin and downstream target proteins in SW480 and DLD1 cells (Fig. 4f and Supplementary Figure S2c). However, the mRNA level of β-catenin was not affected after treatment with PF (Fig. 4g). These data suggested that PF inhibited Wnt/β-catenin signalling by down-regulating the protein level of β-catenin.

### PF promoted the formation of the β-catenin destruction complex and destabilized β-catenin

**Fig. 5:**
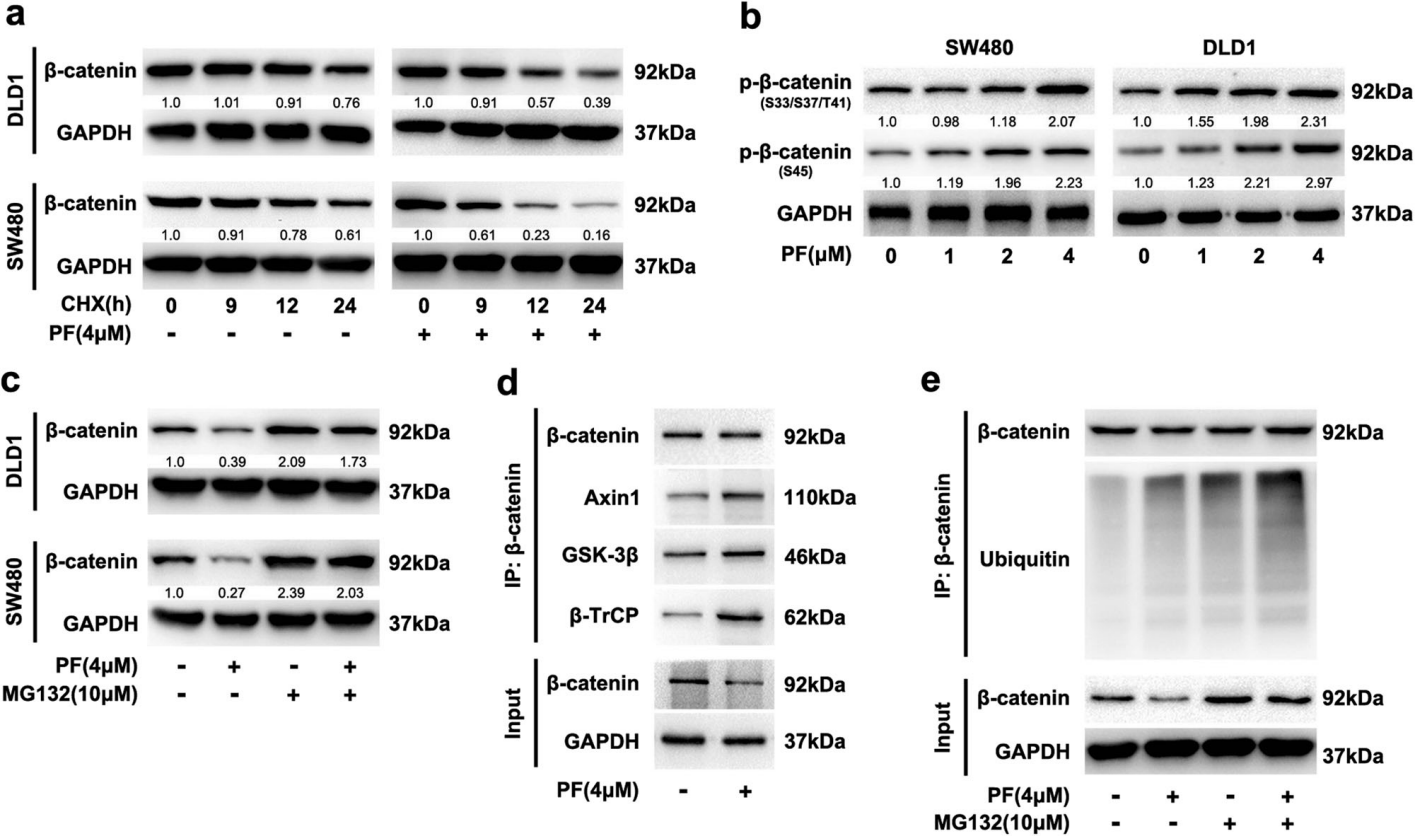
PF promoted the degradation of β-catenin in CRC cells. **a** SW480 and DLD1 cells were incubated with CHX (50 μg/ml) in the presence of PF or DMSO for the indicated time intervals, and the expression of β-catenin was examined by immunoblotting. **b** SW480 and DLD1 cells were treated with indicated concentrations of PF for 24 h, and the intracellular expression of p-β-catenin was analysed by western blot. **c** SW480 and DLD1 cells were incubated with 4 μM PF for 16 h, then with or without 10 μM MG132 for additional 8 h before immunoblotting. **d** Lysates from SW480 cells after treatment with PF (4 μM) was immunoprecipitated with β-catenin antibody, input and immunoprecipitated fractions were analysed by immunoblotting with the indicated antibodies. **e** SW480 cells were incubated with 4 μM PF for 16 h, then with or without 10 μM MG132 for an additional 8 h. The cell lysates were subjected to immunoprecipitation using a β-catenin antibody, and coprecipitating endogenous proteins were detected by western blot with the indicated antibodies

To further determine whether PF promoted the degradation of β-catenin, we investigated the effect of PF on β-catenin stability in the presence of CHX. As shown in Fig. 5a, PF accelerated the degradation of β-catenin in the presence of CHX. Subsequently, we found that PF enhanced the phosphorylation of β-catenin at Ser33/37/Thr41 (Fig. 5b), further suggesting that the degradation of β-catenin was elevated. Intriguingly, the effect of PF on the reduction of β-catenin was remarkably reversed by MG132 (Fig. 5c), indicating that PF enhanced the degradation of β-catenin, which is dependent on the proteasome pathway. Co-immunoprecipitation assays showed that PF facilitated β-catenin binding to Axin1, GSK-3β and β-TrCP (Fig. 5d and Supplementary Figure S3a), suggesting the formation of the β-catenin destruction complex was promoted by PF.

Simultaneously, the ubiquitination of β-catenin was significantly increased after PF treatment, which was more obvious after treatment in combination with MG132 (Fig. 5e and Supplementary Figure S3b). Next, we investigated whether PF affected the interaction of β-catenin and E-cadherin. Co-immunoprecipitation assays revealed that the level of β-catenin interacting with E-cadherin remained unchanged after treatment with PF (Supplementary Figure S3c). These results further demonstrated that PF increased the accumulation of the β-catenin destruction complex and accelerated the degradation of β-catenin, which resulted in inhibition of Wnt/β-catenin signalling.

### PF facilitated YAP binding to β-TrCP and β-catenin destruction complex

**Fig. 6:**
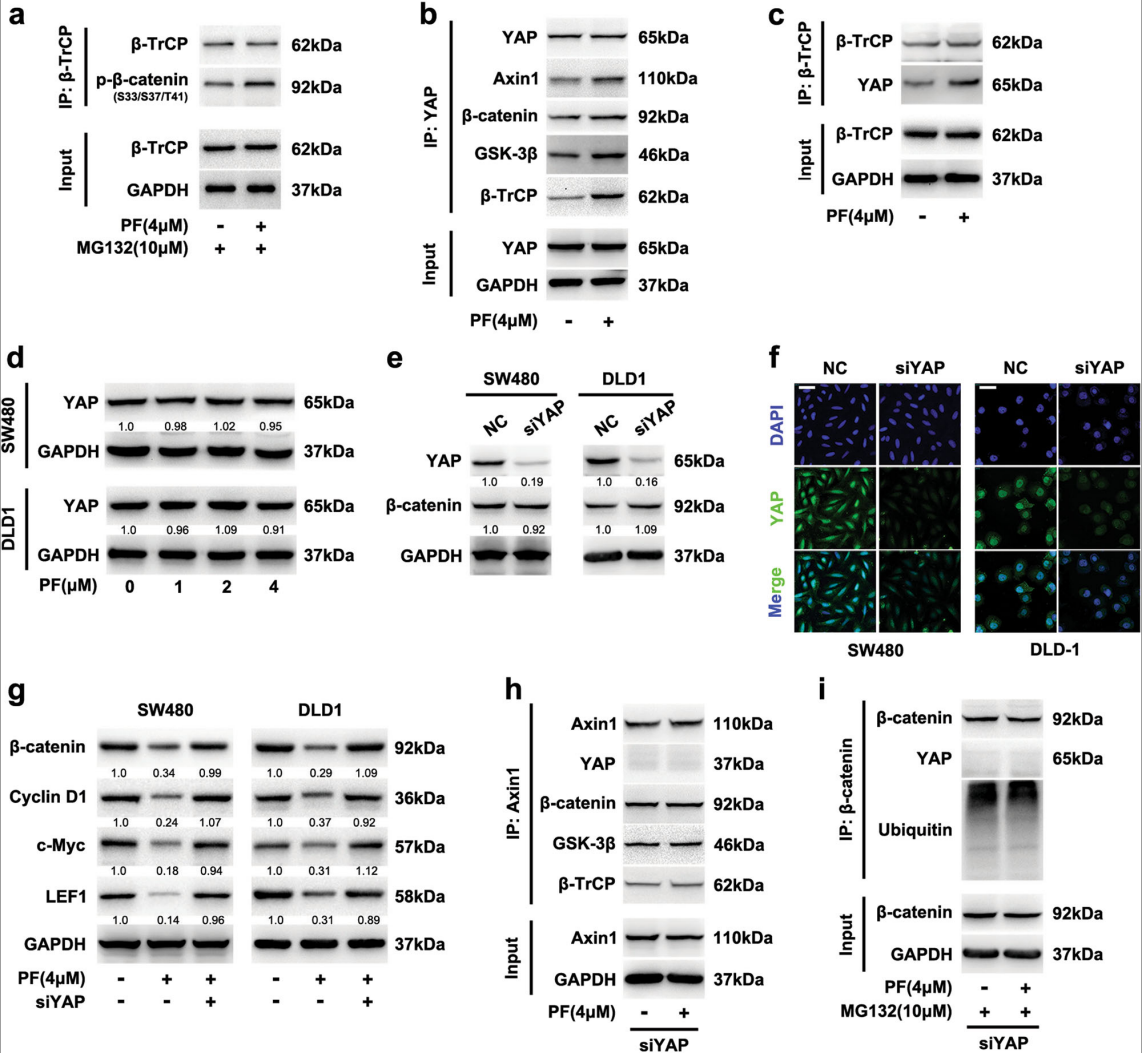
YAP involved in the degradation of β-catenin induced by PF. **a** SW480 cells were incubated with 4 μM PF for 16 h, then with or without 10 μM MG132 for additional 8 h, then cell lysates were subjected to immunoprecipitation using a β-TrCP antibody. **b**, **c** After treatment with PF (4 μM) in SW480 cells for 24 h, cell lysates were immunoprecipitated with YAP (**b**) or β-TrCP (**c**) antibody, then input and immunoprecipitated fractions were analysed by immunoblotting with the indicated antibodies. **d** SW480 and DLD1 cells were treated with different concentrations of PF for 24 h. Then the expression of YAP was determined by western blot. **e** SW480 and DLD1 cells were transfected with YAP siRNA, and the expression of YAP and β-catenin was analysed by immunoblotting. **f** SW480 and DLD1 cells were transfected with YAP siRNA, then the intracellular expression of YAP was analysed by immunofluorescence, scale bars = 20 μm. **g** SW480 and DLD1 cells were transfected with YAP siRNA or negative control, and then incubated with or without PF (4 μM). The expression of β-catenin, cyclin D1, c-Myc and LEF1 was examined by immunoblotting. **h** SW480 cells were transfected with YAP siRNA, then incubated with PF (4 μM) for 24 h, lysates was immunoprecipitated with Axin1 antibody. **i** SW480 cells were incubated with 4 μM PF for 16 h after transfected with YAP siRNA, then with or without 10 μM MG132 for additional 8 h. Cell lysates were subjected to immunoprecipitation by a β-catenin antibody

Accumulating evidences suggest that YAP genetically and functionally interacts with Wnt/β-catenin signalling and is essential for β-TrCP recruitment to the β-catenin destruction complex^12, 14^. To examine the role of PF on the β-TrCP-induced ubiquitination, we investigated the effect of PF on the interaction between β-TrCP and phosphorylation of β-catenin at Ser33/37/Thr41. As shown in Fig. 6a and Supplementary Figure S4a, PF promoted β-TrCP binding to phosphorylated β-catenin at Ser33/37/Thr41. We next analysed whether PF affected the interaction between YAP and β-TrCP. Co-immunoprecipitation assays revealed that PF promoted YAP binding to β-TrCP and the β-catenin destruction complex (Fig. 6b, c, Supplementary Figure S4b and c), while it had no effect on the expression of YAP (Fig. 6d), suggesting that YAP was involved in the PF-induced inhibition of β-catenin.

In addition, tankyrase (TNKS) inhibitors are known to suppress Wnt/β-catenin signalling by stabilizing Axin1 and promoting the formation of the β-catenin destruction complex^25, 26^. We next investigated whether PF could affect Axin1. However, immunoblotting analysis showed that PF did not affect the level of Axin1, while IWR-1 (a TNKS inhibitor)^27^ increased the expression of Axin1 (Supplementary Figure S4d). These findings collectively showed an essential role of YAP in the down-regulation of β-catenin mediated by PF.

### YAP was responsible for PF-mediated inhibition of β-catenin

To further determine whether YAP was contributing to the PF-induced inhibition of β-catenin, we silenced YAP using siRNA and then evaluated the effects of PF on the β-catenin and destruction complex in SW480 and DLD1 cells. After silencing YAP, the expression of YAP was down-regulated (Fig. 6e, f), and the level of β-catenin showed no change, which was consistent with previous studies^28, 29^. Meanwhile, a deficiency of YAP suppressed the proliferation of SW480 and DLD1 cells (Supplementary Figure S5a). More importantly, the inhibition of β-catenin and downstream target proteins mediated by PF were reversed by YAP silencing (Fig. 6g), suggesting that the suppression of β-catenin induced by PF was attributed to YAP.

Co-immunoprecipitation assays showed that YAP deficiency abrogated the formation of the destruction complex induced by PF (Fig. 6h), indicating that YAP was responsible for the PF-induced formation of the β-catenin destruction complex. Concomitantly, the ubiquitination of β-catenin which was increased by PF was also abolished after silencing YAP (Fig. 6i), and similar results were also observed after treatment with verteporfin (VP, a YAP inhibitor) (Supplementary Figure S5b and c). As shown in Supplementary Figure S5d, the proliferation inhibition of SW480 and DLD1 cells mediated by PF were reversed by YAP silencing. Consistent results were also obtained in colony formation assay (Supplementary Figure S5e and f). Collectively, these data indicated that PF inhibited Wnt/β-catenin signalling through accelerating the ubiquitination and proteasome-dependent degradation of β-catenin, which was dependent on YAP binding to the β-catenin destruction complex.

### PF inhibited the growth of xenograft tumours

**Fig. 7:**
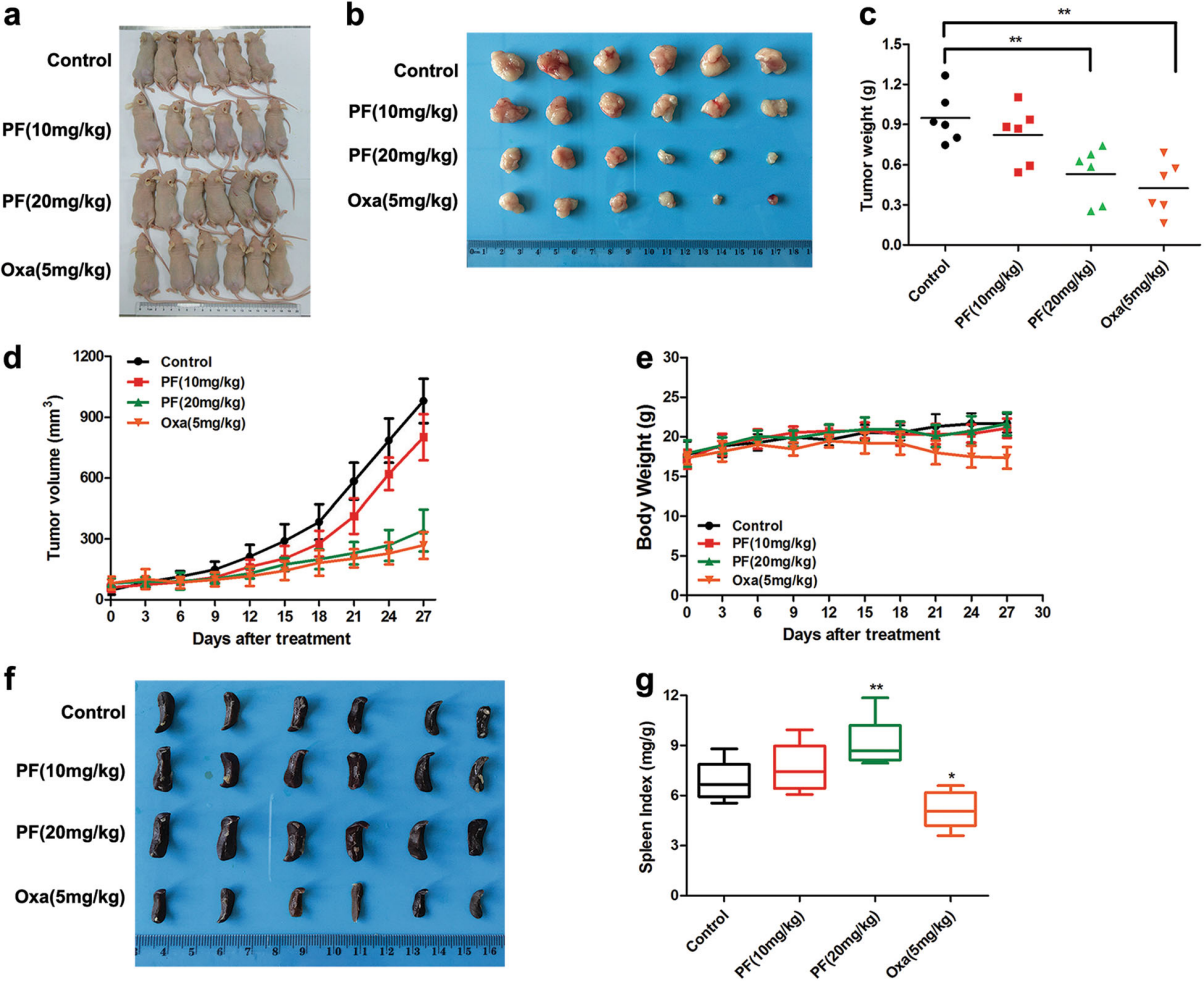
PF inhibited colorectal cancer growth in vivo. **a** Images of SW480 tumour-bearing mice after treated with PF (10 and 20 mg/kg) or oxaliplatin (5 mg/kg). **b–e** SW480 tumour-bearing mice were treated with PF (10 and 20 mg/kg) or oxaliplatin (5 mg/kg) for four weeks. Morphology of tumour (**b**), changes of tumour weight (**c**), tumour volume (**d**), and body weight (**e**) were shown. Data represent the means ± SD (*n* = 6), **P < 0.01, compared with control group. **f** Images of spleen in mice after administration of PF (10 and 20 mg/kg) or oxaliplatin (5 mg/kg) for four weeks. **g** The spleen index was calculated as spleen weight/body weight. Data represent the means ± SD (*n* = 6), *P < 0.05, **P < 0.01, compared with control group

**Fig. 8:**
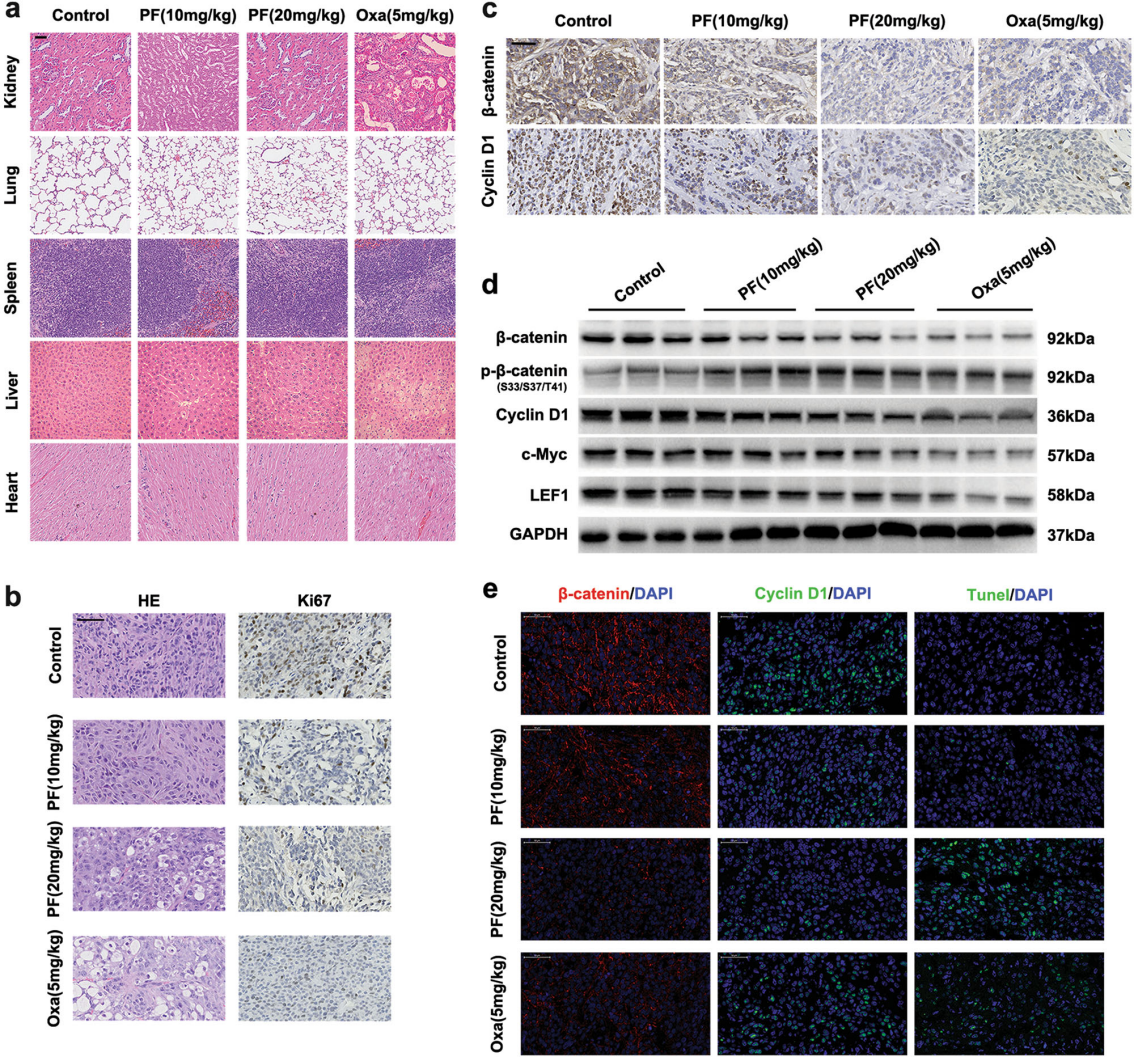
PF decreased the expression of β-catenin in tumour tissue. **a** Hearts, livers, spleen, lungs and kidneys were harvested and sectioned for HE staining. Scale bars = 50 μm. **b** Histopathology of xenograft tumours stained with HE and Ki67. Scale bars = 50 μm. **c** The expression of β-catenin and cyclin D1 was analysed by immunohistochemistry. Scale bars = 50 μm. **d** Tumours were lysed and applied to immunoblotting with indicated antibodies. **e** The expression of β-catenin, cyclin D1 and Tunel in tumours were examined by immunohistofluorescence. Scale bars = 50 μm

To evaluate the anti-tumour capacity of PF in vivo, we established a xenograft model by subcutaneously injecting SW480 cells in nude mice. After the solid tumours reached 100 mm^3^, the animals were randomly divided into four groups. Then, PF and oxaliplatin (Oxa) were intraperitoneally administered every three days. The mice were euthanized after four weeks, and their organs were harvested for histopathologic analysis. As shown in Fig. 7a, b, the growth of tumours was significantly inhibited by PF. Tumour weight and tumour volume were observably decreased after treatment with PF (Fig. 7c, d). Furthermore, PF did not cause an obvious change in mouse body weight (Fig. 7e), whereas the Oxa group showed a body weight loss. Moreover, a remarkable decrease in the spleen weight and spleen index was observed in the Oxa treatment group (Fig. 7f, g). However, the spleen weight and spleen index were increased in the PF treatment group (Fig. 7f, g), suggesting PF had no significant side effects. Pathologically, no obvious morphological changes were observed in the organs of the tumour-bearing mice that were treated with PF, whereas lesions of the kidney and liver were detected in the Oxa treatment group (Fig. 8a). All of these results revealed that PF exerted anti-tumour activity with less toxicity in vivo.

To further confirm the tumour inhibition mechanism of PF, we examined the expression of Ki67, PCNA, β-catenin, Cyclin D1 and c-Myc in the tumour tissue. Immunohistochemistry analysis demonstrated that the expression of Ki67 and PCNA was reduced after PF treatment (Fig. 8b, Supplementary Figure S6a and b), indicating that PF suppressed the proliferation of tumour cells. The expression of β-catenin, Cyclin D1 and c-Myc was also down-regulated in the PF treatment groups (Fig. 8c and Supplementary Figure S6a), which is consistent with the western blot analysis (Fig. 8d). Furthermore, immunohistofluorescence staining of the excised tumour tissue visually revealed that β-catenin and Cyclin D1 were decreased in the PF treatment groups. In addition, the number of tunnel-positive cells was elevated in the PF treatment groups (Fig. 8e), further proving that PF induced the suppression of tumour cell proliferation. Taken together, these results demonstrated that PF exhibited potent anti-tumour activity with an excellent safety profile in vivo.

## Discussion

It is well known that aberrant activation of Wnt/β-catenin signalling is an integral process in the development of most CRC^3, 5^. Increasing evidence strongly suggests that Wnt/β-catenin signalling has emerged as one of the most promising targets for the treatment of CRC^8, 9^.

Withanolides, a class of steroid compounds, exert a wide variety of anti-tumour properties^17^. Although it has been reported that withanolides can inhibit Wnt/β-catenin signalling, the molecular mechanisms remain poorly understood^30^. PF, a withanolide isolated from *Physalis angulata*, exhibited cytotoxicity against a panel of human tumour cell lines^21, 23^. However, the effect of PF on Wnt/β-catenin signalling had not previously been reported. In this study, we demonstrated that PF decreased TOPFlash reporter activity induced by Wnt3a and promoted the proteasomal degradation of β-catenin by accelerating the formation of the β-catenin destruction complex in HEK293T cells.

Previous studies have verified that the β-catenin destruction complex mediates sequential phosphorylation of β-catenin at S45, T41, S37 and S33 for subsequent ubiquitination and proteasomal degradation and thereby maintains low baseline cytosolic levels of the protein in cancer cells^6, 9^. Consistently, we observed that PF inhibited the expression of β-catenin and increased the phosphorylation of β-catenin at Ser33/37/Thr41 in CRC cells. Interestingly, the inhibition of β-catenin was markedly restored by MG132, suggesting the degradation of β-catenin induced by PF was dependent on the proteasome pathway. Because the degradation of β-catenin is mainly regulated by the β-catenin destruction complex^3, 4^, we speculated that PF may affect the formation of the β-catenin destruction complex. Co-immunoprecipitation assays confirmed that PF facilitated the formation of the β-catenin destruction complex and increased the ubiquitination of β-catenin. Moreover, PF had no influence on the interaction of β-catenin and E-cadherin. These data proved for the first time that PF enhanced the degradation of β-catenin through promoting the formation of the β-catenin destruction complex.

YAP, a transcriptional co-activator in Hippo signalling, has been reported to bind to Axin and recruit β-TrCP to the destruction complex^14, 31^, which facilitated the ubiquitination and degradation of β-catenin. To identify whether YAP was involved in the ubiquitination and degradation of β-catenin mediated by PF, we transfected YAP siRNA into CRC cells. After silencing YAP, inhibition of β-catenin induced by PF was eliminated, suggesting that YAP was involved in the PF-mediated inhibition of β-catenin. Meanwhile, PF promoted YAP binding to β-TrCP and formation of the β-catenin destruction complex. Interestingly, YAP deficiency abolished the increase in β-TrCP binding to the destruction complex mediated by PF, indicating that YAP was responsible for the recruitment of β-TrCP to the destruction complex induced by PF. In addition, it has been reported that TNKS inhibitors inhibit Wnt/β-catenin signalling through stabilization of Axin^25, 32^. Unlike TNKS inhibitors, PF did not affect the expression of Axin1. Collectively, our data suggested that the PF-induced inhibition of β-catenin was dependent on YAP binding to the β-catenin destruction complex.

Additionally, a xenograft model bearing SW480 cells was used to evaluate the anti-tumour potency of PF in vivo. Our results showed that PF suppressed tumour growth significantly in tumour-bearing nude mice. Compared with the control group, PF had no significant influence on the animals’ body weight. Importantly, no obvious toxicity was observed in the main organs of mice after treatment of PF, while the Oxa treatment group exhibited remarkable injury to the liver and kidney. Immunohistochemistry and western blot analysis revealed that the expression of β-catenin was decreased in tumours from the PF treatment group. These results further confirmed that PF exerted a remarkable anti-tumour effect with high safety in vivo.

In conclusion, our findings demonstrated for the first time that PF exhibited potential anti-tumour efficacy against CRC in vitro and in vivo by inhibiting Wnt/β-catenin signalling via accelerating the ubiquitination and degradation of β-catenin in a YAP-dependent manner. Our study suggested that PF could be a potential candidate agent for treatment of CRC.

## Materials and methods

### Cell lines and cell culture

HEK293T cells and the human colon cancer cell lines SW480 and DLD1 were purchased from the Cell Bank of Shanghai Institute of Biochemistry and Cell Biology, Chinese Academy of Sciences (Shanghai, China). SW480, DLD1 and HEK293T cells were cultured in DMEM (GIBCO, NY, USA) supplemented with 10% FBS (GIBCO, NY, USA) at 37 °C with 5% CO_2_.

### Cell viability and proliferation assay

A 3-(4,5-dimethylthiazol-2-yl)-2,5-diphenyltetrazolium bromide (MTT) assay was applied to examine cell viability, and relative cell viability was calculated based on the absorbance of untreated cells. Colony formation assays and 5-ethynyl-20-deoxyuridine (EdU) assays were adopted to analyse cell proliferation. For the colony formation assay, cells were seeded into 35 mm plates at a density of 1 × 10^3^/well and incubated overnight. Then, the cells were treated with various concentrations of PF for 24 h. Growth medium was refreshed every three days. After 14 days, the cells were fixed and stained with a crystal violet solution (Sigma-Aldrich, MO, USA) for 15 min, and images of colonies were taken manually.

EdU labelling and detection was performed according to the manufacturer’s instructions (RiboBio, Guangzhou, China). Cells were fixed with 4% paraformaldehyde for 15 min and then washed with PBS and stained with the anti-EdU working solution at room temperature for 30 min. After a wash with 0.5% Triton X-100 in PBS, the cells were incubated with 4,6-diamino-2-phenyl indole (DAPI, Cell Signalling Technology, MA, USA) for 5 min. The cells were observed under ImageXpress® Micro Confocal (Molecular Devices, USA).

### Real-time cell proliferation analysis

The xCELLigence System (Roche Diagnostics GmbH, Mannheim, Germany) was used to monitor the dynamics of the cytotoxic effects of PF on SW480 and DLD1 cells. The xCELLigence System is a microelectronic biosensor system and allows continuous quantitative monitoring of cellular behaviour, including proliferation by measuring electrical impedance^33^. SW480 and DLD1 cells were seeded at 5000 cells/well into E-Plate 16-well plates and cultured overnight. Then, cells were incubated with or without serial dilutions of PF. Cell proliferation was continuously monitored every 15 min over a time period of 60 h. Data analysis was carried out using RTCA Software 1.2.1 supplied with the instrument.

### Luciferase reporter assay

TCF wild-type (TOPFlash) or mutated control (FOPFlash) luciferase reporter plasmids were purchased from Upstate Biotechnology (Lake Placid, NY, USA). Transfection was performed with Lipofectamine 2000 (Invitrogen, Carlsbad, CA) according to the manufacturer’s protocol. Luciferase activity was measured with the Dual Luciferase Reporter Assay System (Promega, WI, USA) according to the manufacturer’s manual. The results were normalized to the control renilla activity.

### Quantitative RT-PCR

Total RNA was isolated using an EASYspin Plus tissue/cell RNA extraction kit (Aidlab Biotechnologies, China). RNA was reverse-transcribed to cDNA using a Transcriptor First Strand cDNA Synthesis Kit (Roche, Basel, Switzerland). Quantitative PCR was performed on a LightCycler 480 system (Roche, Basel, Switzerland) using Fast SYBR Green Master Mix (Roche, Basel, Switzerland). The RT-PCR primers used in this study are listed in Supplementary Table 1.

### Western blot analysis

Cells with different treatments were washed twice with PBS, then collected and lysed in RIPA buffer. The cell lysates were separated on SDS polyacrylamide gels and transferred to PVDF membranes (Bio-Rad, Hercules, CA). After blocking nonspecific binding with TBS-T (0.1% Tween) containing 5% non-fat milk for 1 h at room temperature, the membranes were immunoblotted with the primary antibodies at 4 °C overnight. Then, the membranes were incubated with HRP-conjugated goat anti-rabbit secondary antibody for 2 h at room temperature. The protein bands were detected using the ChemiDOC™ system (Bio-Rad, Hercules, CA). The primary antibodies: Wnt/β-catenin activated targets antibody sampler kit, anti-β-catenin, anti-GSK-3β and anti-YAP were purchased from Cell Signalling Technology (Danvers, MA, USA). Anti-β-TrCP, anti-ubiquitin and anti-Axin1 were purchased from Abcam (Cambridge, UK). HRP-conjugated goat anti-rabbit secondary antibodies were obtained from Cell Signalling Technology (Danvers, MA, USA).

### siRNA transfection

Targeting YAP siRNA was purchased from Biomics (Biomics Biotechnologies, Nantong, China). Cells were seeded in 6-well plates. YAP siRNA was transfected into the cells using Lipofectamine 2000 (Invitrogen, Carlsbad, CA) according to the manufacturer’s instructions.

### Immunofluorescence assay

Cells (1 × 10^4^/well) were cultured on 96-well culture plate (Corning, USA). After incubation with different test substances, the cells were fixed with 4% paraformaldehyde for 15 min, and permeabilized with 0.5% Triton X-100 for 15 min. Next, the cells were blocked with 5% BSA for 1 h and incubated with primary antibody overnight at 4 °C. Alexa-conjugated secondary antibodies (Alexa Fluor 594 goat anti-rabbit IgG, Alexa Fluor 488 goat anti-rabbit IgG, Cell Signalling Technology, MA, USA) were applied and incubated at room temperature for 1 h. Cell nuclei were stained with DAPI (Cell Signalling Technology, MA, USA) for 10 min. Finally, the cells images were analysed by ImageXpress® Micro Confocal (Molecular Devices, USA).

### Immunoprecipitation assay

After treatment, the cells were washed once with PBS and lysed for 15 min on ice. Cell lysates were precleared with the indicated antibody overnight at 4 °C and then with protein A/G beads (Santa Cruz, CA, USA) for another 2 h. After incubation, the protein A/G beads were washed four times with the lysis buffer. The lysates and IP samples were subjected to SDS-PAGE followed by western blot using the indicated antibodies.

### Xenograft tumour model

Five-week-old male BALB/c-nu/nu mice were purchased from the Model Animal Research Center of Nanjing University (Nanjing, China). For the tumour xenograft assay, 5 × 10^6^ SW480 cells were suspended in 200 μl PBS and subcutaneously injected into the right flank of the mice^34^. When the tumours reached approximately 100 mm^3^, the tumour-bearing mice were randomly divided into four groups. Then, PF (10 or 20 mg/kg) and oxaliplatin (Oxa, 5 mg/kg MedChemExpress, NJ, USA) were intraperitoneally administered every three days. After four weeks, all of the mice were euthanized. Then, the tumours and visceral organs of each group were collected and fixed in 4% paraformaldehyde. All animal experimental procedures followed the National Institutes of Health guide for the care and use of laboratory animals and were performed in accordance with protocols approved by the Institutional Animal Care and Use Committee (IACUC) of China Pharmaceutical University Experimental Animal Center.

### Statistical analysis

Statistical analysis was performed with ANOVA or Student’s *t*-test by using GraphPad Prism version 5.0 (GraphPad Software, San Diego, CA). The data were presented as the mean ± SD. *P* < 0.05 was considered significant.

## Electronic supplementary material

Supplementary Tables

Supplementary Figures Legends

Supplementary Figure 1

Supplementary Figure 2

Supplementary Figure 3

Supplementary Figure 4

Supplementary Figure 5

Supplementary Figure 6

Supplementary Author Contribution Form
