# Supplementary material for: YAP-dependent ubiquitination and degradation of β-catenin mediates inhibition of Wnt signalling induced by Physalin F in colorectal cancer
Source: Cell Death Dis. 2018 May 22;9(6):591. doi: 10.1038/s41419-018-0645-3 (PMC5964149; doi:10.1038/s41419-018-0645-3)
Supplement: Supplementary file 1 — Supplementary Tables [file 41419_2018_645_MOESM1_ESM.docx]

**Supplementary Table 1 Primers sequences for RT-PCR analysis**

| Gene | Forward primer (5’-3’) | Reverse primer (5’-3’) |
| --- | --- | --- |
| CTNNB1 | ACAAGCCACAAGATTACAAGAA | GCACCAATATCAAGTCCAAGA |
| c-Myc | GCTGCTTAGACGCTGGATTT | CACCGAGTCGTAGTCGAGGT |
| Cyclin D1 | AATGACCCCGCACGATTTC | TCAGGTTCAGGCCTTGCAC |
| LEF1 | AGGAACATCCCCACACTGAC | AGGTCTTTTTGGCTCCTGCT |
| GAPDH | GCACCACCAACTGCTTA | AGTAGAGGCAGGGATGAT |
