# Supplementary material for: YAP-dependent ubiquitination and degradation of β-catenin mediates inhibition of Wnt signalling induced by Physalin F in colorectal cancer
Source: Cell Death Dis. 2018 May 22;9(6):591. doi: 10.1038/s41419-018-0645-3 (PMC5964149; doi:10.1038/s41419-018-0645-3)
Supplement: Supplementary file 2 — Supplementary Figures Legends [file 41419_2018_645_MOESM2_ESM.docx]

**Supplementary Figure Legends**

**Supplementary Figure 1. PF inhibited Wnt/β-catenin signaling via proteasome-mediated β-catenin degradation.** (**a** and **b**) HEK293T cells were treated with indicated concentrations of PF in the presence of Wnt3a for 16 h, and the expressions of Cyclin D1, c-Myc and LEF1 were examined by immunoblotting (**a**) and qRT-PCR (**b**). Data represent the means ± SD (n=3), **P* < 0.05, ***P* < 0.01, compared with recombinant Wnt3a group, ^##^*P* < 0.01, ^###^*P* < 0.001, compared with untreated group. (**c**) HEK293T cells were treated with 4μM PF for 12 h, then with or without 10μM ALLN and 50nM BTZ for additional 8 h before immunoblotting. (**d**) HEK293T cells were incubated with PF (1, 2 and 4μM) in the presence of BIO (2μM). Immunoblotting assay was performed to observe the protein levels of β-catenin and target proteins. (**e** and **f**) HEK293T cells were treated with 4μM PF for 12 h, followed by addition of DMSO or 10μM MG132 for 8 h. β-catenin was immunoprecipitated with a β-TrCP (**e**) or Axin1 (**f**) antibody. Input and immunoprecipitated fractions were analysed by immunoblotting with the indicated antibodies.

**Supplementary Figure 2. PF suppressed the proliferation of CRC cells.** (**a**) After treatment with indicated concentrations of PF for 24 h, the cell viability was evaluated by MTT assay. (**b**) After treatment with indicated concentrations of PF in DLD1 cells for 24 h, EdU labelling assay was performed and the cells were observed by ImageXpress® Micro Confocal. Data represent the means ± SD (n=3), ***P* < 0.01, ****P* < 0.001, compared with 0μM group, scale bars=50μm. (**c**) SW480 and DLD1 cells were treated with 4μM PF for 24 h, then the intracellular expression of LEF1 was analysed by immunofluorescence. Scale bars=20μm. (**d**) SW480 and DLD-1 cells were treated with the inhibitors of Wnt/β-catenin signaling (IWR-1, VAX-939), then cells were treated with or without PF (4μM), the cell viability was evaluated by MTT assay. ns: no significance. (**e**) SW480 and DLD1 cells were transfected with reporter plasmid, then treated with the inhibitors of Wnt/β-catenin signaling (IWR-1, VAX-939) or PF (4μM), the luciferase activity was measured by the dual luciferase reporter assay system. ns: no significance.

**Supplementary Figure 3. PF promoted the degradation of β-catenin in CRC cells.** (**a**) Lysates from DLD1 cells after treatment with PF (4μM) was immunoprecipitated with a β-catenin antibody, input and immunoprecipitated fractions were analysed by immunoblotting with the indicated antibodies. (**b**) DLD1 cells were incubated with 4μM PF for 16 h, then with or without 10μM MG132 for additional 8 h. Cell lysates were subjected to immunoprecipitation using a β-catenin antibody, and coprecipitating endogenous proteins were detected by western blot. (**c**) After treatment with PF (4μM), cells lysates were immunoprecipitated with a E-cadherin antibody, input and immunoprecipitated fractions were analysed by immunoblotting with the indicated antibodies.

**Supplementary Figure 4. PF promoted YAP binding to destruction complex.** (**a**) DLD1 cells were incubated with 4μM PF for 16 h, then with or without 10μM MG132 for additional 8 h. Cell lysates were subjected to immunoprecipitation using a β-TrCP antibody. (**b** and **c**) After treatment with PF (4μM) in DLD1 cells for 24 h, cell lysates were immunoprecipitated with YAP (**b**) or β-TrCP (**c**) antibody, then input and immunoprecipitated fractions were analysed by immunoblotting with the indicated antibodies. (**d**) SW480 and DLD1 cells were treated with the indicated concentrations of PF or IWR-1 for 24 h, cell lysates were subjected to immunoblotting.

**Supplementary Figure 5. YAP involved in the degradation of β-catenin mediated by PF.** (**a**) SW480 and DLD1 cells were treated with YAP siRNA for 24 h, and cultured for 14 days. The colony formations were assessed by staining with crystal violet. Data represent the means ± SD (n=3), ****P* < 0.001, compared with negative control. (**b**) SW480 cells were co-treated with PF (4μM) and VP (2μM) for 24 h, the cell lysates was immunoprecipitated with a Axin1 antibody, input and immunoprecipitated fractions were analysed by immunoblotting with the indicated antibodies. (**c**) SW480 cells were incubated with PF (4μM) and VP (2μM) for 16 h, then with or without 10μM MG132 for additional 8 h. the cell lysates were subjected to immunoprecipitation by a β-catenin antibody. (**d**) SW480 and DLD1 cells were transfected with YAP siRNA, and then treated with PF for 24 h, the cell viability was evaluated by MTT assay. Data represent the means ± SD (n=3), ***P* < 0.01, compared with PF (4μM) group. (**e** and **f**) SW480 and DLD1 cells were transfected with YAP siRNA, and then treated with PF, colony formation was assessed by staining with crystal violet. Data represent the means ± SD (n=3), **P* < 0.05 and ***P* < 0.01, compared with PF (4μM) group. (**g**) SW480 and DLD1 cells were treated with PF for 24 h, then the cytoplasmic and nuclear fractions lysates were analyzed by immunoblotting.

**Supplementary Figure 6. PF suppressed the proliferation of SW480 cells in vivo.** (**a**) The expression of PCNA and c-Myc was analysed by immunohistochemistry. (**b**) The quantification of PCNA and Ki67 positive cells were measured. **P* < 0.05, ***P* < 0.01, compared with control.
