# Supplementary figures and images for: YAP-dependent ubiquitination and degradation of β-catenin mediates inhibition of Wnt signalling induced by Physalin F in colorectal cancer
Source: Cell Death Dis. 2018 May 22;9(6):591. doi: 10.1038/s41419-018-0645-3 (PMC5964149; doi:10.1038/s41419-018-0645-3)

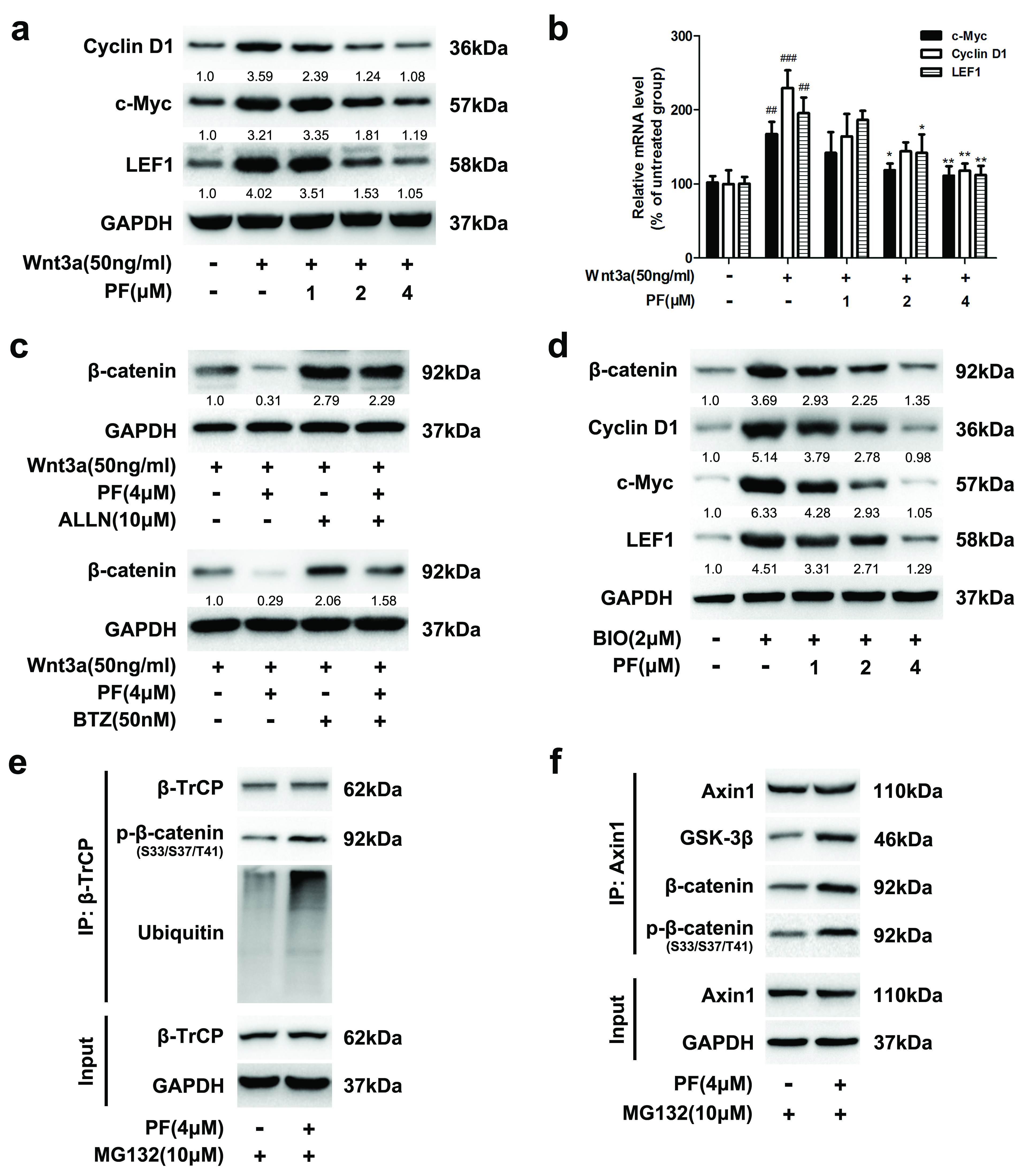

Supplement: Supplementary file 3 — Supplementary Figure 1 [file 41419_2018_645_MOESM3_ESM.jpg]

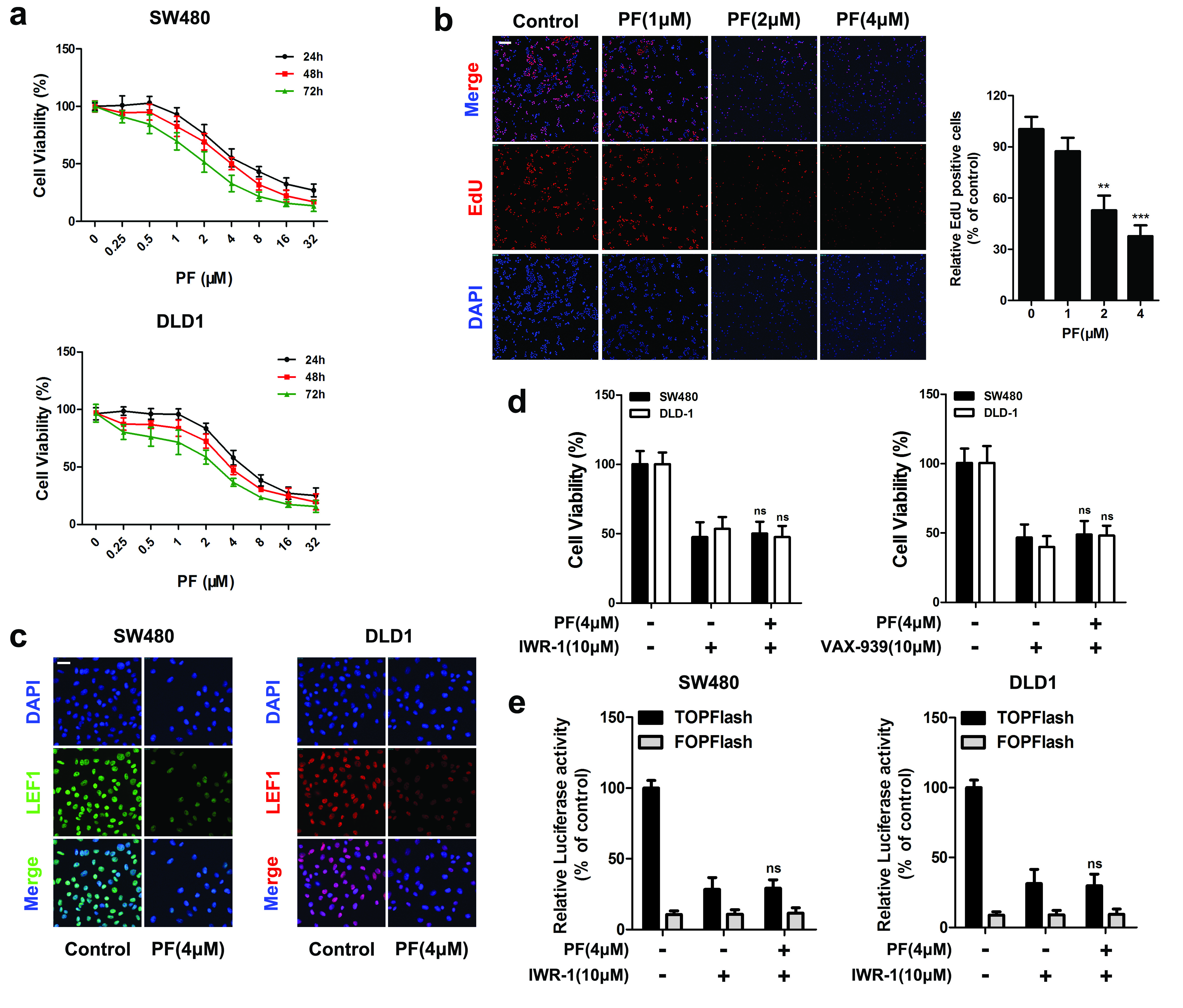

Supplement: Supplementary file 4 — Supplementary Figure 2 [file 41419_2018_645_MOESM4_ESM.jpg]

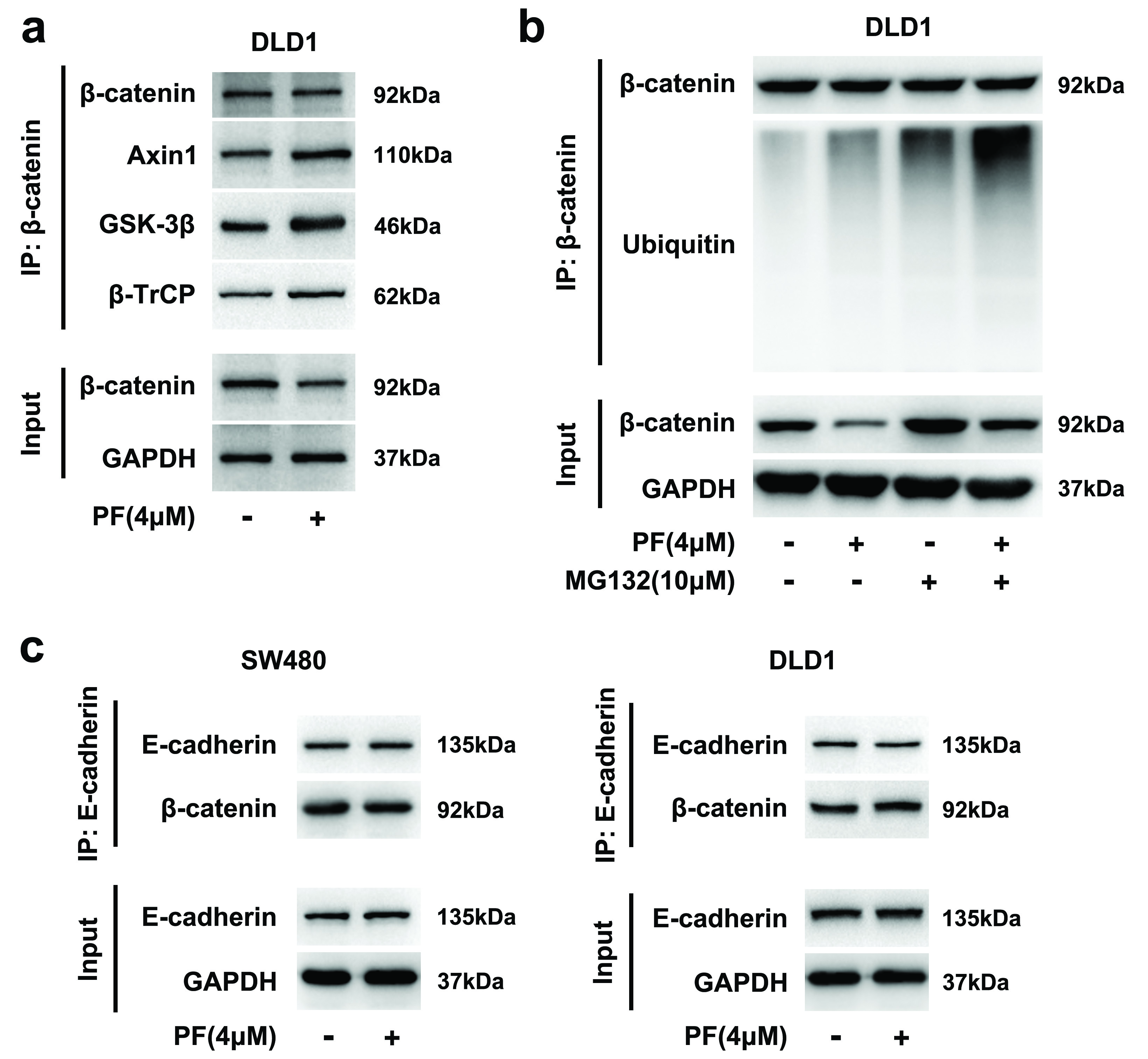

Supplement: Supplementary file 5 — Supplementary Figure 3 [file 41419_2018_645_MOESM5_ESM.jpg]

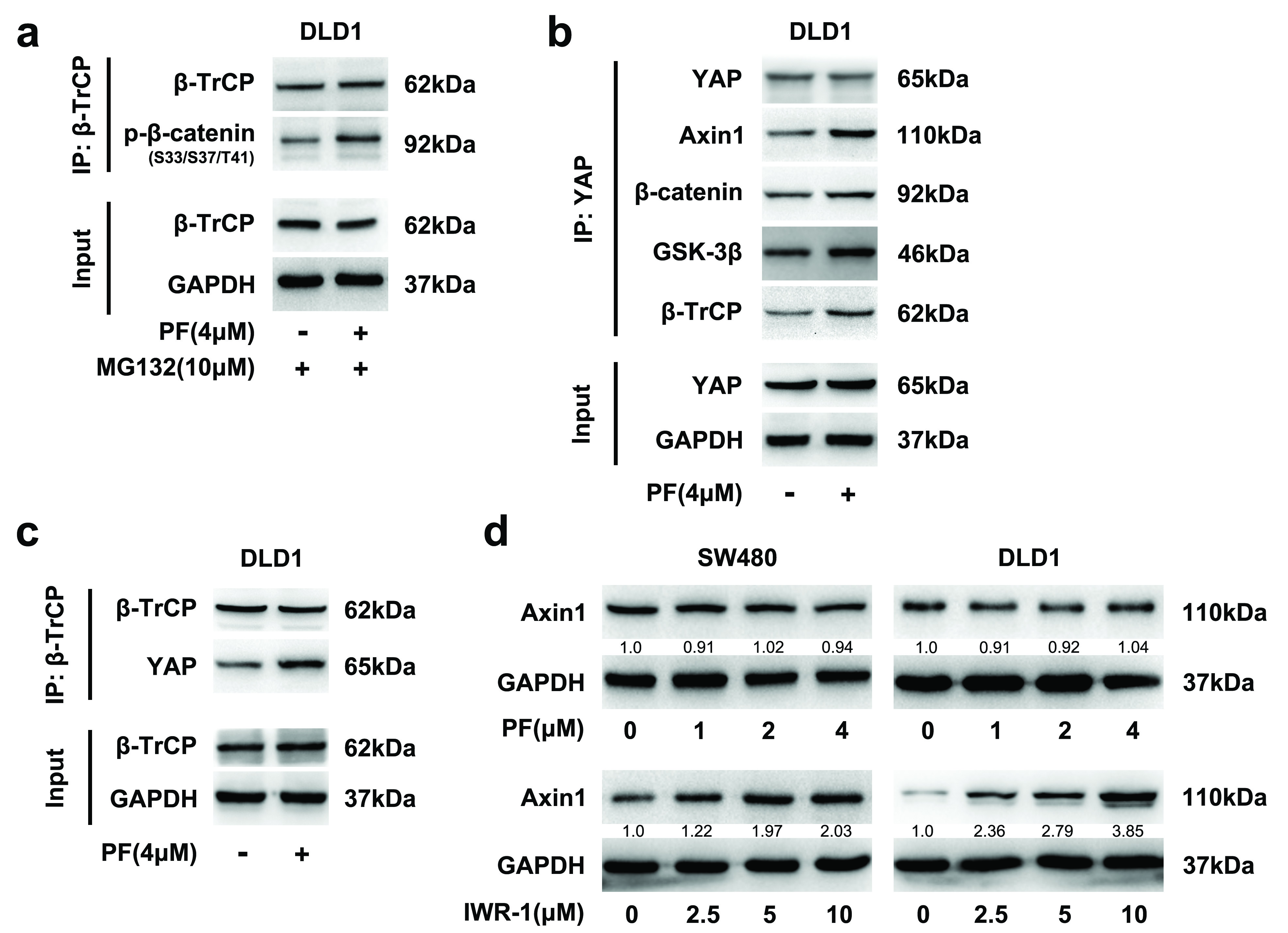

Supplement: Supplementary file 6 — Supplementary Figure 4 [file 41419_2018_645_MOESM6_ESM.jpg]

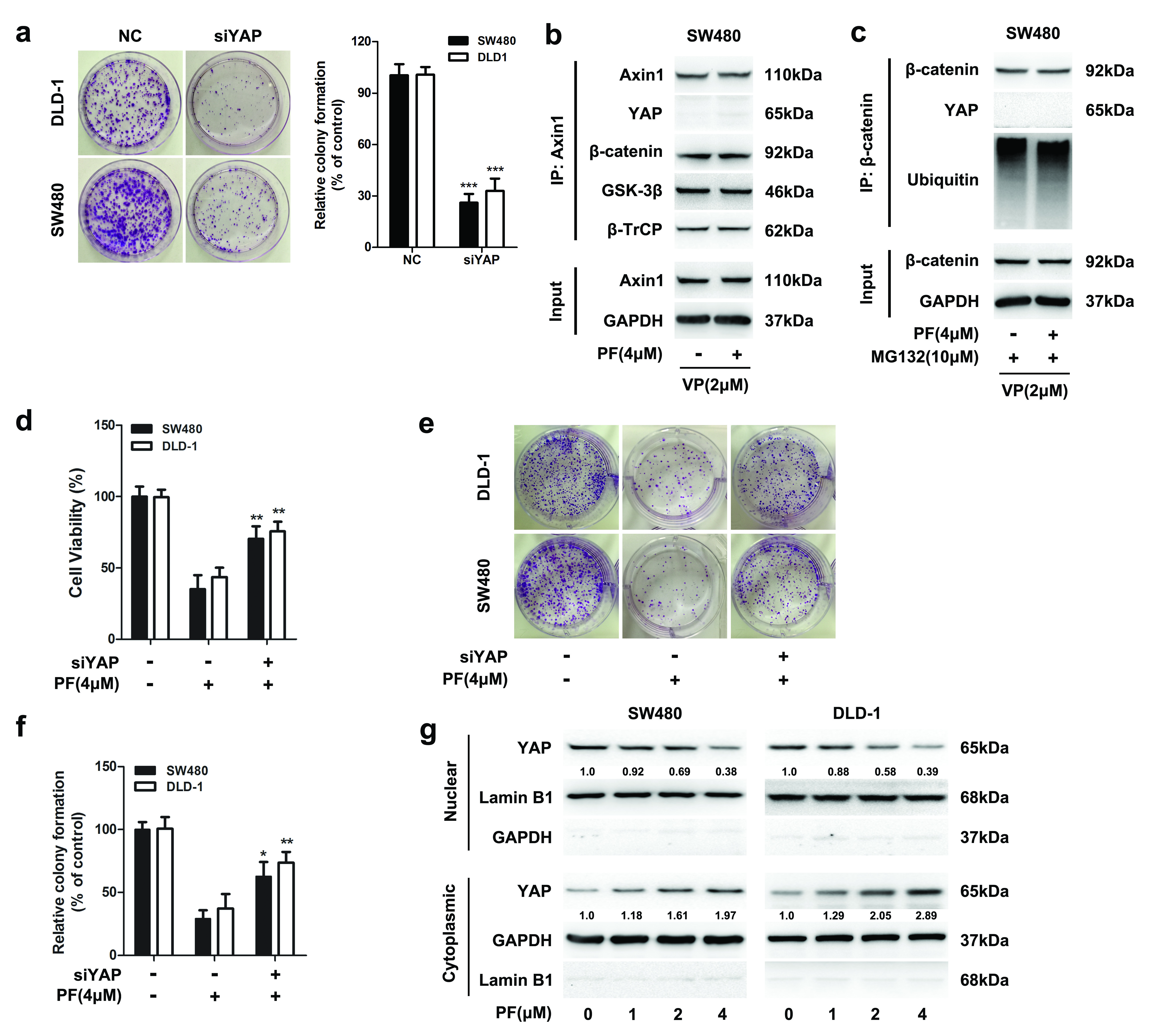

Supplement: Supplementary file 7 — Supplementary Figure 5 [file 41419_2018_645_MOESM7_ESM.jpg]

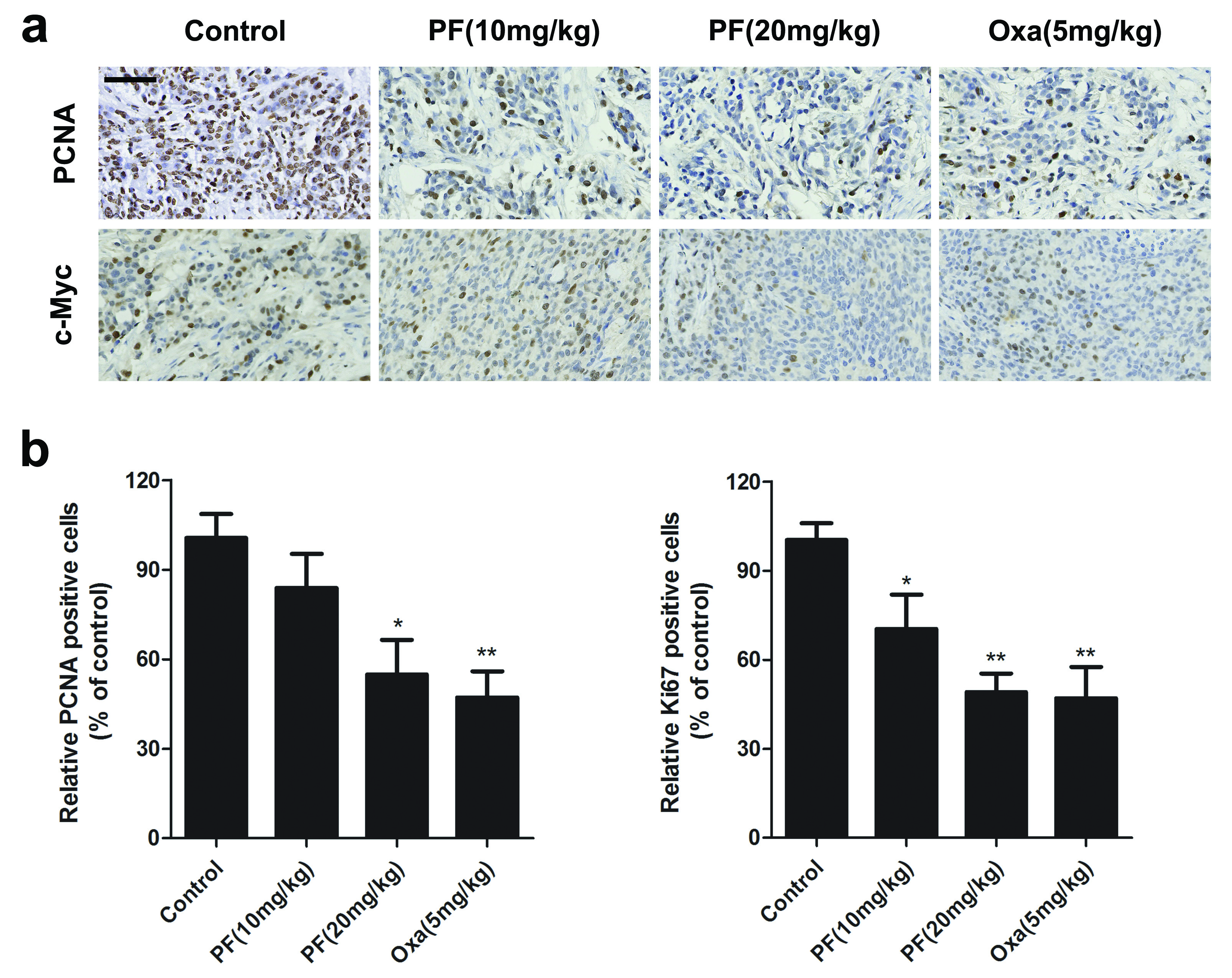

Supplement: Supplementary file 8 — Supplementary Figure 6 [file 41419_2018_645_MOESM8_ESM.jpg]
