# Supplementary material for: YAP-dependent ubiquitination and degradation of β-catenin mediates inhibition of Wnt signalling induced by Physalin F in colorectal cancer
Source: Cell Death Dis. 2018 May 22;9(6):591. doi: 10.1038/s41419-018-0645-3 (PMC5964149; doi:10.1038/s41419-018-0645-3)
Supplement: Supplementary file 9 — Supplementary Author Contribution Form [file 41419_2018_645_MOESM9_ESM.docx]

**1. The contributions of each author**

C.C designed experiments and carried out the experimental work. D.R Zhu helped carry out the experimental work. H.Z provided oversight and helped write the manuscript. C.H helped write the manuscript. G.M Xue helped write the manuscript. T.Y Zhu helped carry out the experimental work. J.G Luo and L.Y Kong conceived the experiments and helped write the manuscript.

**2. The author contributions of each figure**

In Figure 1, C.C generated the data, J.G Luo and L.Y Kong provided compound Physalin F. In Figure 2, C.C generated the data and assembled the figure. In Figure 3, C.C generated the colony formation data, D.R Zhu generated the EdU labeling data. In Figure 4, C.C generated the western blot data, D.R Zhu and T.Y Zhu generated the immunofluorescence data. In Figure 5, C.C generated the western blot and immunoprecipitation data. In Figure 6, C.C generated the western blot and immunoprecipitation data, D.R Zhu and T.Y Zhu generated the immunofluorescence data. In Figure 7, C.C and D.R Zhu generated the xenograft tumour data. In Figure 8, C.C and T.Y Zhu generated the immunehistochemistry and immunofluorescence data.
